# Modelling and comparing the use of IVF and ICSI in Australia

**DOI:** 10.1007/s10815-024-03163-0

**Published:** 2024-06-24

**Authors:** Maria Mazi, Georgina James, Peter Temple-Smith, Ben W. J. Mol

**Affiliations:** Education Program in Reproduction and Development, Department of Obstetrics and Gynaecology, School of Clinical Sciences at Monash Health, Monash University, Clayton, 3168 Australia

**Keywords:** Infertility, Model population, Unexplained infertility, Fertility treatment, IVF, ICSI

## Abstract

**Purpose:**

This study estimates the need of IVF/ICSI in Australia as compared to its actual uptake.

**Methods:**

We created a model estimating for the annual demand for IVF/ICSI in a hypothetical infertile population, using demographic data from medical literature and Australian government databases. For each category of infertility (tubal, severe male, endometriosis, anovulation and unexplained), our estimated need for IVF/ICSI was compared to the actual IVF/ICSI uptake (ANZARD 2019). The model consisted of three categories depending on couples’ cause of infertility, i.e. couples with absolute indications for IVF/ICSI (couples with severe male factor infertility and tubal obstruction); couples with anovulatory infertility (couples with ovulation disorders) and couples with ovulatory infertility (couples suffering from unexplained infertility and endometriosis). The model was applied to each of these categories to determine the number of couples that would require IVF/ICSI treatment after failing to conceive naturally or after following alternative treatment plans. The main outcomes of this study were the estimate of IVF/ICSI cycles and the difference between the estimate and the reported number of IVF/ICSI cycles (2019 ANZARD report).

**Results:**

We estimated that approximately 35,300 couples required IVF/ICSI treatment in Australia in 2019, while in 2019 according to ANZARD, 46,000 couples underwent IVF/ICSI. A higher uptake of IVF/ICSI cycles than expected was specifically reported in couples with unexplained infertility, ovulation disorders and endometriosis, while for tubal and severe male infertility uptake seemed adequate.

**Conclusion:**

In Australia, there seems to be overservicing of IVF/ICSI, specifically for unexplained, ovulatory and endometriosis-related infertility.

## Introduction

In vitro fertilisation (IVF) is the cornerstone of infertility treatment, with Australia being one of the leading countries in the development of assisted reproductive technologies (IVF/ICSI) [1]. IVF was originally introduced in infertile women suffering from tubal pathology. Lesley Brown, the mother of world’s first IVF baby, Louise Brown, had her fallopian tubes removed just before her IVF treatment [2]. The introduction of ICSI allowed treatment of couples suffering from severe male infertility [3]. After these initial successful cases, IVF was also introduced for longstanding infertility of other causes. An early randomised clinical trial confirmed the effectiveness of IVF in women with unexplained infertility for 3 years or longer [4]. Since then, the use of IVF/ICSI has gradually increased, with an exponential increase over the last few decades for various reasons, including social causes, such as later childbearing and an increase in the range and availability of diagnostic tests [5]. Advancing age and ovarian ageing are the most common reasons why clinicians urge women to undertake treatments to enhance and/or preserve their chances of having a baby in the future [6].

During the last decade, infertility treatments have been applied to all types of infertility, including mild male factor, anovulatory and unexplained infertility. This trend has resulted in a continuous increase in IVF treatments, also for couples with a reasonable chance of natural conception [7]. It is important that IVF/ICSI treatment is applied where there is sufficient medical need, and not treating those who can conceive with less invasive methods, thus avoiding potential harm as well as reducing cost of treatment.

It is important for patients with mild endometriosis and unexplained infertility or mild abnormalities not ruling out natural conception to be completely informed by their physicians about their favourable chances for natural conception as well as alternative treatment options, (such as ovulatory hyperstimulation induction (OI) and intrauterine insemination (IUI)), prior to IVF/ICSI treatment.

Recent literature suggests that couples with unexplained infertility have a better chance of becoming pregnant through natural conception than couples with tubal obstruction or severe male factor infertility [8]. Natural conception is also possible in women suffering from endometriosis if the severity of the disease is mild [8]. Moreover, couples suffering from unexplained infertility have a higher chance of spontaneous conception than couples suffering from other causes of infertility [8]. It is important for these patients to be completely informed by their physicians about their favourable chance for natural conception as well as alternative treatment options (such as ovulation induction (OI) and intrauterine insemination (IUI)), prior to IVF/ICSI treatment.

Here, our analysis we aim to explore the use of IVF/ICSI in Australia and to identify situations where more invasive treatments, such as IVF/ICSI, could have been avoided. The aim of the study was to develop a model that examines the use of IVF/ICSI in Australia by comparing the estimated infertile Australian population with the annual number of IVF/ICSI cycles reported.

## Materials and methods

### Outline of the model

A model-based approach was used to estimate the demand for IVF/ICSI using data on medical infertility in Australia. Ethics approval was not required for this study. The model comprised of two main components: (i) an estimation of the number of infertile couples in Australia and (ii) an estimated breakdown of infertility causes within the Australian population. These data provided an estimate of the number of couples annually that would require IVF/ICSI to treat infertility.

### Data collection

For the demographic data collection, the total number of births (and stillbirths) was sourced from national databases and registries for 2019. The Australian and New Zealand Assisted Reproduction Database (ANZARD) was consulted for the number of IVF/ICSI cycles from 2019, as it was the most recent year for which these data were available. We considered the number of births assuming 16% infertility rates to estimate the number of couples confronted with infertility [9, 10] A literature search was conducted to identify key variables between past models and this current model, and to determine the breakdown of infertility causes affecting the Australian population. Keywords including prevalence, infertility and the specific types of infertility indications were searched for the model. The studies were selected based on the similarity of the study population to the Australian population (rebates for fertility treatment and socioeconomic similarities). Variables used in the estimation of Australia’s annual number of infertile couples included the following: infertility prevalence, recurrent miscarriage rate and number of births. We searched Google Scholar and PubMed to identify studies that provided factual data for each of these variables. Variables used in the estimation of IVF/ICSI demand included the following: prevalence of unilateral tubal obstruction, prevalence of bilateral tubal obstruction, prevalence of severe male factor, prevalence of ovulation disorders, prevalence of endometriosis and prevalence of unexplained infertility (Table 1). Additionally, variables used in the breakdown of alternative treatment options included the following: ovulation induction with first-line treatment (clomiphene citrate and letrozole), ovulation induction with second-line treatment (gonadotropin therapy using FSH/laparoscopic ovarian drilling (LOD)), the likelihood of natural conception based on Hunault’s prediction model, expectant management and identification of fertile window and, lastly, intrauterine insemination with controlled ovarian stimulation (IUI-COS) [17, 18].

**Table 1:** The breakdown of infertility causes after fertility workup and treatment success rate sourced from literature

Estimating infertile population	Prevalence (%)	References
Miscarriage rate (conditional on biochemical pregnancy)	20%	(van der Steeg et al. [11]; Verhoeve et al. [12])
Population infertility rate (reproductive age 15–44)	16%	Kamphuis et al. [7]; Newman et al. [10]
Infertility categories based on fertility workup		
Unilateral tubal obstruction	20%	(Robert and Barbieri [13]; Tros et al. [14])
Bilateral tubal obstruction	4%	(Robert and Barbieri [13]; Tros et al. [14])
Severe male factor	15%	(Robert and Barbieri [13])
Endometriosis	5%	Taylor [15]
Unexplained infertility	30%	Snick et al. [16]
Ovulation disorders	26%	Snick et al. [16]
Treatment options for couples with anovulatory infertility		
1st-line treatment success rates (CC/Let)	48%	Falcone and Hurd [17]
2nd-line treatment success rates (gonadotropin therapy/LOD)	32%	Newman et al. [10]
Treatment options for couples with ovulatory infertility		
Spontaneous pregnancy after 12 months (Hunault’s prediction values)	8%	Snick et al. [16]
Expectant management successful pregnancy rate	27%	Gnoth et al. [18]
Intrauterine insemination (IUI) successful pregnancy rate	50%	Newman et al. [10]

### Causes of infertility

Our model was developed using current evidence-based infertility treatment data [15, 16, 19] and was designed to determine the need for IVF/ICSI in Australia. Using these demographic data and the annual IVF/ICSI data for Australia in the 2019 ANZARD report [10], the model estimated the number of infertile couples, based on a 16% infertility rate. A 16% infertility rate also aligns with the conclusions from live births in the 2019 ANZARD report [10] and the infertility rate from a 2006 national fertility study [20]. For the sensitivity analyses, we tested the model using two additional infertility rates of 10% and 20%, based on studies by Snick et al. [16] and Jacobson [19].

Based on the total infertile population that we estimated, three different categories of infertility were defined:

- Couples presenting with absolute indications for IVF/ICSI (couples with unilateral and bilateral tubal obstruction and/or severe male factor infertility)
- Couples presenting with anovulatory infertility requiring IVF/ICSI
- Couples presenting with ovulatory infertility requiring IVF/ICSI

**Fig. 1:**
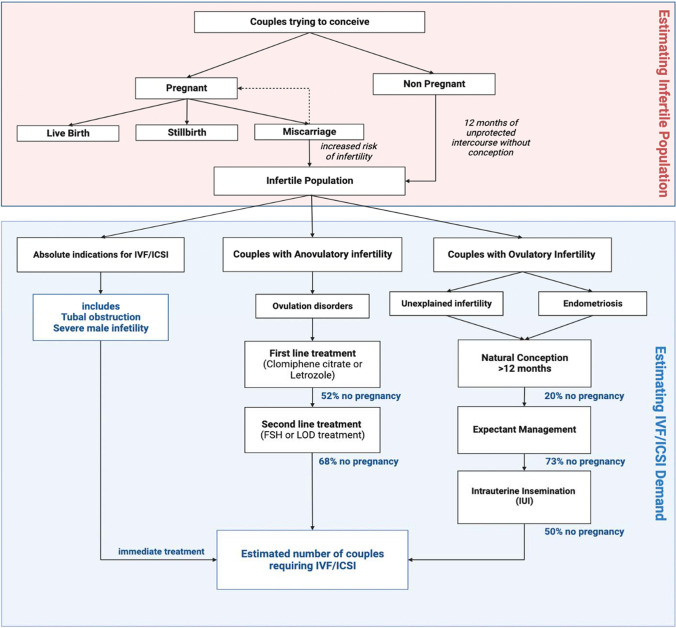
Identifying the infertile population based on national data and determining the need of IVF/ICSI according to a treatment model. Flowchart depicting the calculation process of the model. Initial step included the definition of infertile couples, i.e. couples that failed to achieve pregnancy after 12 months of regular, unprotected intercourse and couples that had two or more pregnancy losses. Infertile population was estimated based on the number of live births, IVF/ICSI live births and number of still births within 1 calendar year. Infertile population was then divided into two categories: anovulatory and ovulatory infertility. Each category included an alternative treatment plan to achieve pregnancy. Couples that were not successful in falling pregnant through the treatment plan were included in the total estimated number of couples that require IVF/ICSI to conceive. The estimate was then compared to the annual ART report [10]

A literature search was conducted to identify the prevalence of the three categories within the total estimated infertile population (16%). These prevalence figures were added together to determine the total number of couples that require IVF/ICSI treatment (Fig. 1). This estimated number was then compared to the reported number of couples had undergone IVF/ICSI in Australia in 2019.

Based on the estimated number of couples, we calculated the estimated number of cycles for each infertility category by multiplying the number of couples by 2, as ANZARD data indicates that the average number of cycles undertaken by each couple in 2019 was 1.8 [10]. Our estimated number of cycles for each category was then compared to the number of cycles of the infertility categories used by ANZARD for data collection [10]. The estimated number of cycles conducted for absolute indications was compared to the sum of two equivalent ANZARD categories—tubal obstruction and severe male factor. The estimated number of cycles for ovulatory infertility was compared to the reported cycle numbers for unexplained infertility and endometriosis. The estimated number of cycles for anovulatory infertility was compared to the sum of the equivalent categories (ovulation disorders, other female factors, combination of female factors). In the 2019 ANZARD report, “other female factors” includes infertility caused by fibroids, ovulation disorders or premature ovarian failure [10]. Our analysis did not include calculations for patients with a combination of multiple female factors or with a combination of female and male factors. Couples with unexplained infertility were also not included in this study. The calculations of this model were based on the average age of first-time mothers in Australia in 2019, which was stated at 29.4 [21]. As the average age is below 35 years of age, for couples suffering from ovulatory infertility, we assumed that the course of treatment would follow the steps displayed on Fig. 1.

## Results

### Estimating the need for IVF/ICSI in Australia

In 2019, there was a total of 305,832 live births and out of those, 15,158 were IVF/ICSI live births. The total infertile population in Australia in 2019 was calculated to be 40,766 couples [10, 22, 23].

Table 2 shows the breakdown of infertility causes and the estimated number of cycles receiving IVF/ICSI (and number of cycles conducted) treatment of 16% infertility rate, excluding the number of couples with a combination of female and male causes. Comparison of the data from ANZARD and the outputs of our model show an excess of IVF/ICSI treatments using each of the three different infertility rates in couples suffering from both ovulatory and anovulatory infertility. The results also showed that fewer cycles were conducted for couples with absolute indications than what it may be expected based on the results of our model (Table 2).

**Table 2:** Infertility category results and total estimated and reported infertile population in Australia in 2019 with 16% infertility rate

Indication for IVF/ICSI	Estimated number of infertile couples	Estimated needed number of IVF/ICSI cycles*	Performed number of IVF/ICSI cycles				
Absolute indications			Fresh		Thawed		Total
Tubal obstruction	16,727	33,454	1462		1188		2650
Severe male factor	10,455	20,910	5539		3888		9427
Anovulatory infertility							
Ovulation disorders (**other female factors)	6408	12,816	13,092	7121		20,213	
Combination of female factors	N/A	N/A	4018	2833		6851	
Combination of male and female causes	N/A	N/A	3471	2533		6004	
Ovulatory infertility							
Endometriosis	254	508	2634	1617		4251	
Unexplained infertility	1526	3052	8440	5828		14,268	
Not stated cause	N/A	N/A	12,797	7620		20,417	
Total	35,370	70,740	51,453	32,628		84,081	

*Our analysis estimated the total number of IVF/ICSI cycles in 2019, including both fresh and thawed cycles.**Other female factors include infertility caused by fibroids, ovulation disorders or premature ovarian failure [10]. Our analysis did not account for combination of female factors or a combination of male and female factors affecting fertility

ANZARD indicates that 46,073 couples underwent IVF/ICSI treatment in 2019 in Australia and the reported number of IVF/ICSI cycles was 84,081 in Australia and New Zealand. The infertility rate was adjusted to 10% and 20% [15, 19] for the sensitivity analysis (Tables 3 and 4 in the Appendix).

### Sensitivity analyses

**Fig. 2:**
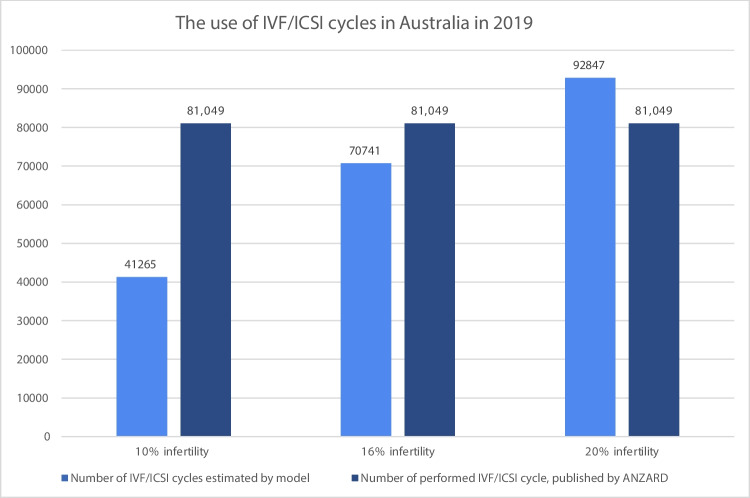
A comparison of the combined estimated need of IVF/ICSI and the reported use of IVF/ICSI in Australia in 2019 (including fresh and frozen/thawed), according to 10%, 16% and 20% infertility rate [10, 16, 19]

Figure 2 shows a comparison between the estimated and the reported treatment cycles for IVF/ICSI in Australia in 2019 using the 16% infertility rate and for the 10% and 20% infertility rates used in the sensitivity analysis.

## Discussion

This study developed a prognostic model that estimated the demand for IVF and ICSI treatment in Australia. Prognostic models are used to help clinicians provide the best possible treatment option to infertile couples and to enable the government to assess appropriate use of health funding [24]. While the demand for fertility treatment is clear for couples with known infertility, (such as tubal obstruction and severe male factor infertility), it is often suggested that couples suffering from anovulatory (ovulation disorders) or ovulatory infertility (mild endometriosis or unexplained infertility) should explore alternative treatment options before proceeding to more advanced procedures such as IVF and/or ICSI.

Our model showed an overuse of IVF/ICSI treatments in Australia when assuming a 10% or 16% infertility rate but an underuse for an infertility rate of 20%. Moreover, the analysis showed an overuse of IVF/ICSI cycles in couples suffering from unexplained infertility, ovulation disorders and endometriosis. Possible explanations of this increased number of cycles in the unexplained infertility category may include women of advanced maternal age accessing fertility treatment or ovulatory disorders due to irregular periods as a sign of ageing. Additionally, the model showed that couples suffering from tubal obstruction or severe male infertility are under-treated as the performed number of IVF/ICSI cycles is lower than the estimated number of IVF/ICSI cycles. It is common that not all patients undergo complete laparoscopic testing prior to accessing fertility treatment, leading to many patients suffering from tubal obstruction to be reported as “unexplained infertility”. Although it can be argued that this overuse is supported by a goal to provide couples with a family while reducing waiting time, careful consideration of the risks associated with IVF/ICSI procedures should be given when proceeding to treat couples with IVF/ICSI that have a favourable prognosis for natural conception [25].

Research examining the benefits and harms associated with infertility treatment has shown that the rise of IVF/ICSI is driven by the focus of achieving a live birth (and subsequently forming a family) while often overlooking its potential health and social impact on both the children and the mother [1]. Despite the potential unknown issues, couples are often advised to continue their IVF/ICSI journey even though less advanced and alternative treatment options can be made available for consideration before proceeding to IVF/ICSI. Our model was developed according to previous prediction and prognostic models that highlight the importance of natural conception and alternative treatments.

Prognostic tools and national guidelines are available to guide physicians in deciding whether, and when, a couple will require IVF/ICSI or when they would benefit from waiting and try alternative treatment options before opting for more advanced IVF/ICSI treatments, if the woman is younger than 35 years old. Research shows that prediction models can be clinically applied during fertility workup to provide the couples with their best chances for a pregnancy [26]. Prognostic tools exist online and can be accessed by the public; examples of prediction models include the YourFertilityTool from Fertility Coalition in Australia and the tool from the Dutch patient organisation Freya [27, 28]. These online resources are readily available, easily to use and are important educational devices for patients to estimate their chance of spontaneous conception.

### Accessibility to IVF-related medical rebates

Although Australia has mostly private fertility clinics, IVF treatment is more accessible than in other countries due to the widely subsidised system. In the Netherlands, public funding for fertility treatment includes an unlimited number of IUI cycles and three IVF or ICSI cycles [29]. Australians are eligible to claim rebates for their fertility treatment, varying from $400 to $3200 from the government, through Medicare [30–32]. The model identified that the number of IVF/ICSI cycles is lower in the group of women suffering from tubal obstructions than the estimated number of cycles calculated by the model. Several questions arise surrounding this finding, such as the accessibility of IVF/ICSI treatment for these couples or the lack of specific gynaecological investigation prior to treatment. To understand this finding, more holistic studies need to be conducted.

### Limitations of the model

The calculations in the model, even though this can be seen as a limitation, were developed using assumptions obtained from literature that provided similar trends across a variety of studies [10, 16, 19].

Moreover, a limitation of the model is that it does not include infertility due to multifactorial causes. In most reports, the distribution of infertility causes includes the combination of fertility complications, including combined male and female factor infertility, a combination of male factor and endometriosis as well as other combinations of causes. Unfortunately, the ANZARD report does not include a breakdown of the combination of infertility causes; thus, we could not adjust this in our model. By including an estimate of the number of couples with a combination of causes for infertility, the calculated number of couples, requiring IVF/ICSI treatment, would be lower than the numbers presented in this project. A third limitation for this study is that the ANZARD report is a collective of the data from Australian and New Zealand IVF clinics that provides an aggregate for the number of couples or women undergoing IVF/ICSI treatment, as approximately 10% of the total number of cycles were conducted in New Zealand. Additionally, the model does not include moderate male factor, where alternative therapies could be used to treat low sperm count. There is potential for the model to be adjusted according to data provided in future ANZARD reports to include same-sex couples and single mothers seeking fertility treatment as well as mild forms of male infertility [10].

The purpose of this study was to examine the use of IVF and ICSI in Australia, in comparison to an estimated number of couples and cycles that require IVF/ICSI to treat infertility. To improve clinical pathways that direct the use of IVF/ICSI, restrictions to treatment and government funding policies for IVF/ICSI need to be revisited and restructured in order to provide appropriate treatments for infertile couples in Australia.

## Appendix

For the number of total live deliveries, birth data included total number of births from single and multiple gestations. The annual number of pregnant women over 20 weeks of gestation, not attributable to IVF/ICSI births, from total deliveries, stillbirths and IVF/ICSI deliveries.

To identify the total number of non-IVF/ICSI l.

$$\begin{array}{l}\text{Non}-\text{IVF}/\text{ICSI pregnancies}\\ =\text{number of total live deliveries}-\text{number of IVF}/\text{ICSI deliveries}\\ +\text{number of stillbirth}\end{array}$$

From this, we calculated the fertile population, using 20% miscarriage rate:

$$\text{Fertile population}=\text{number of non}-\text{IVF}/\text{ICSIpregnancies divided by }1-\text{miscarriage rate}$$

We then estimated the annual number of couples planning pregnancy from our fertile population and the infertility rates used, to conduct a sensitivity analysis.

$$\text{Population planning pregnancy}=\text{ertile population divided by }1-\text{infertility rate}.$$

Lastly, from our population planning pregnancy, we determined the annual number of infertile couples at our baseline infertility rate (10%) and sensitivity analysis (16%).

$$\text{Total infertile population}=\text{population planning pregnancy}\times \text{infertility rate}$$

### Estimating the Demand for IVF/ICSI

The total infertile population was divided into three categories, according to the type of infertility diagnosed. Couples that presented unilateral or bilateral tubal obstruction as well as severe male factor infertility were identified as couples with absolute indications for IVF/ICSI treatment.

$$\begin{array}{l}\text{Couples with absolute indications for IVF}/\text{ICSI}\\ =\text{total infertile population}\times (\text{unilateral tubal obstruction prevalence}\\ \begin{array}{c}+\text{bilateral tubal obstruction prevalence}\\ +\text{severe male factor infertility prevalence})\end{array}\end{array}$$

$$\begin{array}{l}\text{Couples with absolute indications for IVF}/\text{ICSI}\\ =\text{total infertile population}\times (\text{unilateral tubal obstruction prevalence}\\ \begin{array}{c}+\text{bilateral tubal obstruction prevalence}\\ +\text{severe male factor infertility prevalence}\end{array}\end{array}$$

### Anovulatory infertility

Couples that were diagnosed with ovulation disorders were classified as couples with anovulatory infertility and underwent hormonal treatment prior to IVF/ICSI.

$$\text{Couples with anovulatory infertility}=\text{total infertile population}\times \text{ovulation disorder prevalence}$$

### Ovulatory infertility

Couples that were diagnosed with ovulatory infertility included couples with unexplained infertility and endometriosis.

$$\text{Couples with endometriosis}=\text{total infertile population}\times \text{endometriosis prevalence}$$

$$\text{Couples with unexplained infertility}=\text{total infertile population}\times \text{unexplained infertility prevalence}$$

### Estimated number of infertile couples in Australia

The number of couples requiring IVF/ICSI in Australia in 2019 is the sum of couples with absolute indications, couples with anovulatory infertility with unsuccessful pregnancies after hormonal treatment and couples with ovulatory infertility with unsuccessful pregnancies after IUI (Tables 3 and 4).

**Table 3:** Infertility category results and total estimated and reported infertile population in Australia in 2019 with 10% infertility rate

Indication for IVF/ICSI	Estimated number of infertile couples	Estimated needed number of IVF/ICSI cycles*	Performed number of IVF/ICSI cycles		
			**Fresh**	**Thawed**	**Total**
Tubal obstruction	9758	19,516	1462	1188	2650
Severe male factor	6099	12,198	5539	3888	9427
**Anovulatory infertility**					
Ovulation disorders (**other female factors)	3738	7476	13,092	7121	20,213
Combination of female factors	N/A	N/A	4018	2833	6851
Combination of male and female causes	N/A	N/A	3471	2533	6004
**Ovulatory infertility**					
Endometriosis	148	296	2634	1617	4251
Unexplained infertility	890	1780	8440	5828	14,268
Not stated cause	N/A	N/A	12,797	7620	20,417
Total estimated number of couples needing IVF/ICSI	20,633	**41,266**	51,453	32,628	**84,081**

*Our analysis estimated the total number of IVF/ICSI cycles in 2019, including both fresh and thawed cycles.**Other female factors include infertility caused by fibroids, ovulation disorders or premature ovarian failure Newman et al. [10]. Our analysis did not account for combination of female factors or a combination of male and female factors affecting fertility

**Table 4:** Infertility category results and total estimated and reported infertile population in Australia in 2019 with 20% infertility rate

Indication for IVF/ICSI	Estimated number of infertile couples	Estimated needed number of IVF/ICSI cycles*	Performed number of IVF/ICSI cycles			
			**Fresh**	**Thawed**		**Total**
Tubal obstruction	21,955	43,910	1462	1188		2650
Severe male factor	13,722	27,444	5539	3888		9427
**Anovulatory infertility**						
Ovulation disorders (**other female factors)	8410	16,820	13,092		7121	20,213
Combination of female factors	N/A	N/A	4018		2833	6851
Combination of male and female causes	N/A	N/A	3471		2533	6004
**Ovulatory infertility**						
Endometriosis	334	668	2634		1617	4251
Unexplained infertility	2003	4006	8440		5828	14,268
Not stated cause	N/A	N/A	12,797		7620	20,417
Total estimated number of couples needing IVF/ICSI	46,424	**92,848**	51,453		32,628	**84,081**

*Our analysis estimated the total number of IVF/ICSI cycles in 2019, including both fresh and thawed cycles.**Other female factors include infertility caused by fibroids, ovulation disorders or premature ovarian failure Newman et al. [10]. Our analysis did not account for combination of female factors or a combination of male and female factors affecting fertility
